# Characteristics of person, place, and activity that trigger failure to speak in children with selective mutism

**DOI:** 10.1007/s00787-021-01777-8

**Published:** 2021-04-24

**Authors:** Christina Schwenck, Angelika Gensthaler, Felix Vogel, Anke Pfeffermann, Sabine Laerum, Julia Stahl

**Affiliations:** 1 Department of Special Needs Educational and Clinical Child and Adolescent Psychology, Justus-Liebig-University of Giessen, Otto-Behaghel-Straße 10c, 35394 Giessen, Germany; 2 Department of Child and Adolescent Psychiatry, Psychosomatics and Psychotherapy, University Hospital Frankfurt am Main, Frankfurt, Germany; 3 Department of Linguistik/Patholinguistik, University of Potsdam, Potsdam, Germany

**Keywords:** Selective mutism, Qualitative research, Online study, Triggers for failure to speak

## Abstract

Selective Mutism (SM) is an anxiety disorder with predictable and circumscribed situations in which children remain silent while they speak unaffectedly in others. However, core features of anxiety inducing stimuli have rarely been studied so far. Parents of children with elevated SM symptomatology participated in an online-based study and answered open ended questions about specific characteristics of a person, place, and activity that elicit failure to speak in their child. The final sample consisted of *n* = 91 parents with children aged between 3 and 17 years (*M* = 8.02 years, *SD* = 3.94). Answers were analyzed by qualitative content analysis. Characteristics of a person were assigned to five categories with lack of distance as the most frequently reported feature. With respect to a place, the majority of parents mentioned unknown places as a silence trigger. The most frequently mentioned feature of an activity that was designated to be associated to silence was new activity. There were only few associations between the designation of these features, age, and gender. For the first time, anxiety inducing triggers related to person, place, and activity were comprehensively assessed in children with SM. This allows a differentiated and deeper understanding of an understudied disorder. The majority of characteristics can be associated with proposed etiological factors such as increased behavioral inhibition, conditioning processes, social anxiety, and a strong need for control. Implications for effective treatments are discussed.

## Introduction

Selective Mutism (SM) is a mental disorder with a typical onset in early childhood. According to the fifth edition of the Diagnostic and Statistical Manual of Mental Disorders (DSM-5), it comprises symptoms of consistent failure to speak in certain social situations that include an expectation for speaking behavior [1]. These situations must be circumscribed and predictable. At the same time, children with SM show unimpaired speaking behavior in other situations. Epidemiological studies indicate a mean prevalence rate of about one percent [2]. Therefore, the disorder cannot be described as rare, as it occurs at least about as often as autism spectrum disorder. However, compared to other mental disorders in childhood and adolescence, SM is highly underdiagnosed and understudied. Higher prevalence rates have been found in bi-/multilingual children [3, 4]. The disorder is associated with the child’s severely impaired psychosocial functioning, and both, social and educational development are typically influenced considerably [5]. Additionally, the few longitudinal data available so far indicate that SM is not a temporary state that resolves by itself [6, 7]. In combination with the severe impairments caused by this disorder, a need for scientific findings that provide insights into the disorder and from which adequate treatment approaches can be derived becomes evident.

In DSM-5, SM was classified among anxiety disorders for the first time. This decision was based on a significant number of studies indicating anxiety as a central phenomenon of SM, a common etiology between SM and other anxiety disorders, and results from initial treatment studies [4, 8, 9]. High comorbidity rates between SM and other anxiety disorders, in particular with social anxiety disorders (SAD), have been found [9–11]. Etiologically, behavioral inhibition (BI) was found to display a significant role for anxiety disorders in general and for SM in particular. This temperament trait includes features of shyness, distress to novelty, and fear [12]. Early BI has been found to be a strong predictor for later SAD [13–15]. According to a retrospective evaluation by their parents, children with SM were rated as to be more behaviorally inhibited than children with SAD, children with internalizing disorders and typically developing children [16]. Other etiological factors discussed for SM are classical and operant conditioning [2] and reduced auditory processing in a smaller subgroup of children with SM [17–20]. Lastly, the few randomized controlled trial treatment studies available so far indicated promising results for behavioral/cognitive-behavioral treatments [2]. Interestingly, some of the successful treatment approaches added aspects of defocused communication and child directed interaction to the core features of behavioral treatments [21–24]. Defocused communication is characterized by taking direct attention away from the child, e.g., having the therapist sit next to the child instead of opposite from him, and establishing joint attention. In child directed interaction, the therapist lets the child take the lead, praises appropriate behavior, reflects what the child is doing, or imitates appropriate play.

Despite the definition of SM by the DSM-5, which requires the predictability of silence, the core features of anxiety inducing stimuli for children with SM have rarely been studied empirically yet. On the contrary, SM is the only anxiety disorder for which the DSM-5 does not specify anxiety eliciting triggers [1]. Clinically, three major factors influencing speaking behavior of children with SM have been identified, specifically the *person* with which the child interacts, the *place* where the interaction happens and the *activity* that is undertaken. Because a social situation is usually influenced by all three factors an interaction between the three components can be assumed and children are usually confronted with more than one anxiety eliciting stimuli [25]. The three factors were confirmed by a factor analysis of a screening questionnaire [26] although another screening tool has found only one factor on which all items loaded [27]. Empirical data on talking patterns of children with SM are sparse yet, but there are some single research results that indicate proof for the existence of such factors that have an influence on the speaking behavior of children with SM.

With respect to the *person*, unfamiliar people seem to trigger silence in the vast majority of children with SM [7, 28]. Some children with SM remain silent in the contact with other children in general, while others only cannot talk to specific children [7]. Additionally, the teacher frequently displays a person with whom children with SM cannot speak [29], and children with SM were found to remain silent more frequently in contact with adults compared to other children [28]. On the contrary, most children with SM speak with the members of their core family like parents and siblings, and familiarity has a positive influence on speaking behavior [7, 30]. With regard to the *place* of speaking behavior, most evidence indicates that children with SM remain silent in the school setting and day care while they usually speak unimpaired at home [7, 28–30]. Furthermore, public and new settings, family gatherings, and social events were found to be associated to muteness in children with SM [30]. Regarding the *activity*, being the focus of attention and being involved in stressful activities were found to have a negative influence on the ability to speak of children with SM [30].

In a quasi-experimental study that compared children with SM, SAD and typically developing children aged eight to 18 years, different categories of video scenes around the school setting were presented. Results indicated that children with SAD experience social evaluative situations as more fear eliciting as situations with speech demands, while children with SM rate both type of situations alike [31]. However, the situations presented in this study contained interacting triggers of person, place, and situation. It is therefore impossible to conclude which specific triggers were experienced by participants as causing anxiety. In a qualitative study children and adolescents aged 8 to 18 years were asked for the fear content and related cognitions responsible for remaining silent. Here, participants mentioned social fears regarding negative evaluation by others, fears of mistakes and language/voice related fears to be responsible for their muteness [20].

Despite of this insight in factors that have an influence on speaking patterns in children with SM, it must be noted that the majority of studies that addressed this question so far assessed the factors with closed question format and therefore might have missed important aspects of person, place and situation that were not asked for. Furthermore, no study assessed all three aspects at the same time allowing for a concise overview. The two studies that addressed the question more comprehensively only included children from the age of eight years. Because the mean age of onset for SM lies in preschool years, it remains unclear whether developmental aspects have an influence on the triggers for mute behavior. Therefore, the aim of the current research project was to assess characteristics of person, place, and activity with open ended questions and the adoption of qualitative evaluation of the answers given. With the help of an online survey, parents of children with elevated SM symptomatology participated in the current study, resulting in a comparably huge sample size and wide age range. The data available to date indicate familiarity of person, place, and activity to have an influence on speaking behavior in children with SM. Furthermore, an influence of circumscribed conditioned places with negative experiences such as school on silence has been described. Regarding the activity, activities which contain attention and evaluation by others, activities with speech demands, and challenging activities with the possibility to fail were suggested to influence the speaking behavior of children with SM negatively. Due to the inductive approach of the qualitative analysis, no concrete hypotheses were formulated.

## Materials and methods

### Sample

Initially, *n* = 224 parents started the online-survey, of which *n* = 127 finished it. Of these 127 participants, *n* = 36 did not exceed the cut off for SM in a diagnostic measure for the screening of SM [27]. Therefore, our final sample consisted of *n* = 91 parents of children and adolescents (*n* = 68 female) with elevated SM symptomatology (SM). Inclusion criteria comprised an age of the child below 18 years as well as a score above the threshold for SM symptomatology.

### Procedure

All parents participated in an anonymous online-based study conducted with the help of UNIPARK software. The survey was advertised through different media such as mental health professionals, inpatient and outpatient clinics, newspapers, online-forums, and schools. Initially, parents were informed about the study, and informed consent was given by button press. All parents answered a questionnaire about general information and standardized questionnaires. Then, parents were asked open ended questions that were anxiety eliciting for their child and would lead to silence. The study was approved by the local Ethics Committee of the Faculty of Psychology and Sports Science of the University of Giessen (Germany).

### Assessment

#### *Frankfurt Scale of Selective Mutism (FSSM)* [27]

The Frankfurt Scale of Selective Mutism (FSSM) is a parent-rated questionnaire developed to screen for and evaluate Selective Mutism in children aged between three and 18 years. So far, the scale is freely available in German, English, Norsk, and Suomi (forward–backward translated). There are different versions for kindergarteners aged 3 to 7 years, schoolchildren aged 6 to 11 years, and teens aged 12 to 18 years. The questionnaire consists of a Diagnostic Scale with ten yes–no questions about the children’s overall speaking behavior. The Severity Scale comprises 41 or 42 questions regarding the specific speaking behavior at kindergarten/school, in public, and at home that are answered on a 5-point-Likert-Scale. An evaluation of the FSSM revealed excellent reliability scores (*α* = 0.90–0.98) and validity with a one-factor solution for the Severity Scale [27]. The diagnostic scale differentiates excellently between children with SM and a combined group of children with SAD, internalizing disorders and control children with sensitivities of 94–97% and specificities of 90–95% (AUC = 0.97–0.99) [27]. In the current study, we applied the sum score of the Diagnostic Scale (range 0–10) and the Severity Scale’s relative score (range 0–1). The age-appropriate version (FSSM 3–7, FSSM 6–11 or FSSM 12–18) for each participant was applied. Only data sets were included in the study if the cut-off value for SM in the respective age group was exceeded in the diagnostic scale (see Gensthaler et al. [27]).

#### Open ended questions

All parents were introduced to the relationship between fear and mute behavior and then presented with three open-ended questions about the characteristics of a person, a place, and an activity that causes anxiety and mute behavior in their children (e.g. *“Please indicate which characteristics of a person/place/activity your child finds anxiety-provoking.”*). Parents could answer these questions in a text field without word limitation and indicate as many answers as they wanted. No examples were given so as not to influence parents’ response behavior.

### Data analysis

A qualitative content analysis (QCA) was applied to each of the three open ended questions with the aim to classify answers into content categories [32, 33]. Following this procedure, all answers were read to get an overall impression in a first step. Then, text units that contained aspects of significant characteristics of a person/place/activity were highlighted and headings for these passages were formulated. These headings were then grouped into higher order categories, and a description of each category was formulated. Text units were then assigned to the different categories. Frequencies with which aspects of the different categories were named were calculated, and for each participant it was coded whether a certain category had been mentioned or not (1/0 coding), because parents could indicate as many aspects as they wanted. Finally, to assess inter-rater-reliability, an independent and blind researcher assigned all answers to the prescribed higher-order categories.

The statistical package for the social sciences (SPSS 24) was used to assess statistics about sample characteristics. Furthermore, Pearson correlations were calculated for the relationship between categories and age as well as severity of SM symptomatology. If the categorial variable is coded with 0 versus 1, Pearson correlation equals point-biserial correlation. For the relationship between categories and bi-/multilingualism (0 = no, 1 = yes) and gender (0 = male, 1 = female) Phi-coefficient is indicated. Because of the exploratory nature of correlation analysis correction for multiple testing was not undertaken.

## Results

### Sample characteristics

All children were aged between three and 17 years (*M* = 8.02 years, *SD* = 3.94, *MD* = 6.00 years), 42.9% attended nursery school, 54.9% school, and 2.2% neither nor. Seventy-four children had been diagnosed with SM before, 15.4% were currently receiving psychotherapeutic care, and *n* = 2 children were treated with selective serotonin reuptake inhibitors. The sample comprised *n* = 12 children who were raised bilingual, and 67% of these bilingual children were born in Germany. In 20.9% of all families there was one parent with migration background, and in 3.3% of the families both parents were born in a country other than Germany. With 47.3% the majority of participants reported to have a monthly net family income between 3.000 and 4.999€, 38.5% indicated to have between 1.000 and 2.999€. Thus, an upper medium socioeconomic status of the sample can be assumed on average. The mean sum of the Diagnostic Scale of the FSSM was *M* = 8.93 (*SD* = 1.124), and the mean relative score of the Severity Scale was *M* = 0.704 (*SD* = 0.122).

### Open ended questions

Overall, 86.8% of the participants gave at least one codable answer across all three questions. There were no differences in age, gender, or SM symptom severity between parents who gave a codable response or those who did not. Within the sample, participants differed with respect to the number of indicated categories of anxiety inducing characteristics related to a person (*M* = 1.22, *SD* = 0.917, Min = 0, Max = 4), place (*M* = 0.90, *SD* = 0.746, Min = 0, Max = 2) and activity (*M* = 0.98, *SD* = 0.830, Min = 0, Max = 4).

#### Anxiety inducing characteristics of person

Answers from *n* = 69 parents were categorized, the remaining *n* = 22 parents had either not answered the question or had given an answer that did not match the question (“Avoidance of eye-contact”) or that reflected an unawareness of characteristics (“I don’t know”). Within the answers of the 69 parents, 151 codable units were identified. With the help of QCA, five broader categories were extracted which are demonstrated in Table 1.

**Table 1:** Categories of reported anxiety inducing person-characteristics

Category	Description	Examples	Participants who reported this characteristic*	Characteristic compared to all reported characteristics**
Lack of distance	People who do not keep the distance, who get too close to the child physically; too directly address the child or demands and expectations that put pressure on the child; show little sensitivity to the child’s need for distance	“Demanding people” “People who do not keep enough distance” “People who put her under pressure”	45%	28%
Authority characteristics	Behavior and characteristics of a person usually perceived as authoritarian or aggressive or group belonging to authorities	“Strict persons” “Loud voice” “Dominant persons” “Medical doctors”	36%	25%
Low familiarity	Strangers whom the child does not know and who are difficult for him to assess and unpredictable	“Strangers” “Not seen for a long time” “When it hardly knows the person”	33%	16%
External characteristics	Externally visible or audible per se neutral characteristics of a person	“Old age” “Male” “Tall”	25%	21%
Little child-focused	People who are not very focused on the needs of the child and show little sensitivity in the sense of too much distance or clumsy contact with the child, who do not try to get access to the child or who are insensitive to contact	“Closed people” “Unrelaxed and stiff” “Unfriendly”	22%	11%

*Only participants who had reported any codable characteristic (*n* = 69); **n = 151 codable units

Of all parents who gave a codable answer, 45% indicated a characteristic of a person that fell into the category *lack of distance*. This category comprised descriptions of people that do not show sensitivity to the child’s need for distance physically or psychologically, who address the child too directly or put pressure on the child by demands and expectations. About one third (36%) of the parents gave at least one answer that fell into the category *authority characteristics*. This category summarized behaviors and characteristics of a person that are usually perceived as authoritarian or aggressive. Also, about one third of the parents described features of *low familiarity* (33%), such as strangers and people who are difficult to assess or unpredictable to the child. Lastly, about one fourth of the parents gave an answer that fell into the category *external characteristics* (25%) and *little child-focused behaviors* (22%). The first category comprised visual or audible external characteristics which usually are perceived as neutral by the general population such as height, hair color, or high-pitched voice. The latter category included features of people that show little sensitivity in the sense of too much distance, clumsy contact with the child or people that do not try to connect emotionally with the child.

Inter-rater reliability of these categories was high with Kappa coefficient of *κ* = 0.914.

#### Anxiety inducing characteristics of place

With respect to anxiety inducing characteristics of a place, answers from *n* = 61 parents were categorized. Within the answers of these parents, 92 codable units were identified. With the help of QCA, four broader categories were extracted which are demonstrated in Table 2.

**Table 2:** Categories of reported anxiety inducing characteristics of place

Category	Description	Examples	Participants who reported this characteristic*	Characteristic compared to all reported characteristics**
Unknown places	Unknown places that the child does not yet know or only knows a little, which are associated with uncertainty with regard to procedures and little behavioral safety	“Places that are new for my child” “If this is the first time anywhere” “Unknown place”	56%	46%
Crowds	Places with a lot of people	“When there are too many people in one place” “Many people in little space” “Many people”	44%	29%
Places with negative experience	Places where the child has already had negative experience or expects to meet or talk to certain people	“Medical practice” “Places where she is expected to speak” “Negative experience at this or similar place”	21%	16%
High volume	Places with high volume or much noise	“Volume dominates” “Noisy environment” “Loud noises”	13%	9%

*Only participants who had reported any codable characteristic (*n* = 61); **n = 92 codable units

More than half of the parents (56%) gave at least one answer that fell into the category *unknown places*. This category comprised places that the child is unfamiliar with or which are associated with uncertainty with regard to procedures and little foreseeability in order how to behave correctly. Furthermore, 44% of parents indicated that places with a lot of people (category *crowds*) were anxiety inducing. About a fifth of the parents (21%) stated that *places with negative experiences* displayed places of fear. The category comprised places where the child had already had negative experiences or where it expected to meet certain people or expectations to talk. Lastly, 13% of the parents indicated that places with *high volume* or lots of noise caused fear in their children.

Inter-rater reliability of these categories was high with Kappa coefficient of *κ* = 0.935.

#### Anxiety inducing characteristics of activity

With respect to anxiety inducing characteristics of activities, answers from *n* = 64 parents were categorized. Within the answers of these parents, 89 codable units were identified. With the help of QCA, five broader categories were extracted which are demonstrated in Table 3.

**Table 3:** Categories of reported anxiety inducing characteristics of activity

Category	Description	Examples	Participants who reported this characteristic*	Characteristic compared to all reported characteristics**
New activities	Activities that the child does not yet know, where he/she does not know what to expect and where the consequences are unforeseeable	“Everything that is new” “What he does not know and cannot judge” “The unknown activity”	47%	29%
Motor activities	Motor activities to be learned, activities that require courage or could be potentially dangerous	“Climb up somewhere” “Movements/activities considered to be dangerous” “Swimming, skating and other activities where he could lose control”	27%	17%
Failure	Activities that the child cannot do or is afraid of failing and has not yet mastered	“When she has to do something and is not sure if she can or can’t do it” “Has previously had negative experiences with it” “When she feels overwhelmed”	25%	21%
Focus of attention	Activities where the child could be the focus of attention	“When many people are watching” “When she is observed by strangers” “If she attracts the attention of others in the process”	22%	18%
Activities with speech demands	Activities associated with talking to other people	“Something to talk about” “Speech required” “If you want her to speak in front of others”	19%	15%

*Only participants who had reported any codable characteristic (*n* = 64); **n = 89 codable units

Of all parents who gave a codable answer, 47% indicated that a *new activity* would induce anxiety to their child. This category included activities that the child does not yet know and where the child does not know what to expect. About a fourth of the parents indicated that *activities associated with failure* (25%) and *motor activities* (27%) were anxiety inducing to their child. The first category comprised activities that the child cannot do or is afraid of failing and has not yet mastered, while the second category included motor activities and activities that could be potentially dangerous. Furthermore, 22% of the parents mentioned activities where the child could be the *focus of attention*, and 19% indicated that *speech demanding activities* would be anxiety provoking for their child.

Inter-rater reliability of these categories was acceptable with Kappa coefficient of *κ* = 0.841.

### Correlations with child characteristics

For each of the three open ended questions, the fit of each category was correlated with age, SM symptom severity, bi-/multilingualism, and gender of the child. Results can be seen in Table 4.

**Table 4:** Pearson correlations/ Phi-coefficients between age, SM symptom severity, bi-/multilingualism, gender and fit of category

Target	Category	Age	SM symptom severity (FSSM)	Bi-/multilingualism	Gender
Person	External characteristics	0.104	0.122	− 0.081	− 0.025
	Authority characteristics	0.182	− 0.021	− 0.054	0.178
	Low familiarity	− 0.087	0.142	− 0.136	0.075
	Little child-focused	− 0.203^+^	− 0.047	− 0.061	− 0.148
	Lack of distance	− 0.196	0.062	0.179	− 0.089
Place	Unknown places	− 0.279*	− 0.166	− 0.188	− 0.061
	High volume	− 0.268*	0.131	0.249^+^	− 0.272*
	Crowds	− 0.048	0.093	0.188	0.141
	Places with negative experience	0.312*	0.067	0.122	− 0.022
Activity	New activities	− 0.320**	− 0.100	− 0.146	0.224^+^
	Activities with speech demands	0.079	− 0.007	0.014	0.077
	Failure	− 0.128	− 0.119	− 0.050	− 0.106
	Focus of attention	0.151	0.240^+^	0.189	0.025
	Motor activities	− 0.027	0.212^+^	− 0.064	− 0.085

*FSSM* Frankfurt Scale of Selective Mutism

^+^*p* < 0.10; *p < 0.05; **p < 0.01

With respect to characteristics of a person, there was only a negative trend between age and the category *little child focused* (*r* = − 0.203, *p* = 0.095) indicating that the older a child with SM was, the less frequently caregivers identified distant and clumsy behavior of a person as an important feature for their child’s silence. With regards to characteristics of a place, there were negative correlations between age and the categories *unknown places* (*r* = − 0.279, *p* = 0.029) and *high volume* (*r* = − 0.268, *p* = 0.037), and a positive correlation with *places with experiences* (*r* = 0.312, *p* = 0.014). Furthermore, there was a trend for a correlation between the category *high volume* and bi-/multilingualism (*φ* = 0.249, *p* = 0.052). The same category correlated negatively to gender (*φ* = − 0.272, *p* = 0.034) indicating that high volume is more relevant for boys with SM and less for girls. Finally, regarding characteristics of an activity, there was a negative correlation between the category *new activities* and age (*r* = − 0.320, *p* = 0.010) and a positive trend with this category and gender (*φ* = 0.224, *p* = 0.073) indicating that parents of girls tended to mention this category more frequently than parents from boys. Furthermore, there were positive trends for the association between SM symptom severity and the categories *focus of attention* (*r* = 0.240, *p* = 0.056) and *motor activities* (*r* = 0.212, *p* = 0.093)*.* All effect sizes are considered small except from medium effect sizes for the associations between age and *places with experiences* and age and *new activities*.

## Discussion

The current study was designed to identify characteristics of the variables person, place, and activity that have an influence on the speaking behavior in children with elevated SM symptomatology. We chose a qualitative research strategy to ask parents about specific characteristics of these variables in an online survey.

In line with past research results regarding characteristics of a *person* as an interaction partner we had expected familiarity [7, 28] to display a crucial role for speaking behavior of children. Indeed, *low familiarity* was reported to be an important characteristic that prevents children with SM symptomatology from speaking. Behavioral inhibition has been found to display an important role for the etiology of SM [16], and distress to novelty is one core aspect of this temperament feature [12], which might explain for the strong reactivity of children with SM towards low familiarity. However, less than a quarter (22%) of the parents mentioned this feature spontaneously and it represented only 11% percent of all codable units. In contrast, the most frequently mentioned category was *lack of distance* with almost half of the parents indicating this feature to be associated with their child’s silence. This characteristic has not been directly described to be associated with mutism in children yet. However, defocused communication and child directed interaction have been found to be important aspects in successful behavioral treatments [21–24]. These specific procedures take away any pressure to talk from the child and allow him to maintain the necessary distance and control over what is happening throughout the therapeutic process. Thus, they specifically address the increased need of many children with SM for control and less demanding social interaction. This finding might also explain the fact that some children with SM verbally interact with selected children or adults while they remain silent in interaction with others [7]. This supports the clinical experience, that being an unfamiliar person even may play into the clinicians’ favor. This is true at least, if the clinician is able to connect with the child in a way that is completely stripped off any pressure to speak at first. Children with SM tend to rigidly and consistently divide their world into the people, places, and activities that are associated with either being able to talk or not being able to talk. Once the speaking-pattern is established and a person is assigned the role of a non-talking-person, it is more difficult for the child to overcome the silence than to start talking with a new person whom the child does not have a history of silence with. People on the non-talking side are said to be “contaminated”. Lack of distance in the behavior of the interaction partner could still cause the child to feel that his scope for action is limited and that the situation is inescapable. Such situations can cause a freeze response which is a passive coping strategy [34]. Among others, freeze response is expressed in immobility, including motor and vocal inhibition [35], which are typical symptoms in children with SM.

In line with some data indicating that many children with SM have difficulties talking to their teacher [29], and that they less frequently talk to adults than to children [28] we found *authority characteristics* to display an important role for silence with 36% of the parents mentioning this category. Again, this finding may reflect the fear component of the temperament feature behavioral inhibition [16]. Lastly, about a quarter of the parents mentioned certain external characteristics like an old age or body size and little child focused behavior like low interest in the child or hesitant and stiff interaction to be a reason for their child’s silence. Even though the external characteristics mentioned comprised a wide range of very different and contradictory features between children, they are constant within children and therefore predictable as required by DSM-5 [1]. This finding may also indicate classical conditioning to display an etiological mechanism in SM. Since presenting little child focused behavior seems to play a role for a quarter of children, it would be reasonable to review formal requirements, e.g. the child needing to be present during the processing of formalities that are more likely to be carried out with the caregivers. During the diagnostic interview the clinician and the caregiver will usually speak about the issues a child has around his talking behavior. Instead of building up a playful and meaningful relationship with the child, the first contact with the child is stamped with little child focused behavior. Interestingly, there were no correlations of either category with age, SM symptom severity, bi-/multilingualism, or gender. Therefore, categories with respect to person do not seem to be specific for certain subgroups or sensitive to development and experience with the disorder.

With regard to the *place* of speaking behavior, most studies so far described the school setting and day care as locations where children with SM remain silent [7, 28–30]. Therefore, we had expected to find circumscribed places with negative experience to have an influence on speaking behavior of children with SM symptomatology. However, only a fifth of the parents (21%) indicated that *places with negative experiences* are crucial for the silence of their children, and these places not only comprised the school setting but also other places like medical practice or any place where the child had experienced speech demands in the past. The school setting may have been too evident for some parents to mention explicitly, and the result underlines the importance of trans-situational measurement of the symptomatology which is in line with previous studies. It also indicates that previous experiences make it more difficult for the child to overcome silence, a fact that displays an important indication for the treatment of children with SM as initial silence in the therapy setting should be avoided in order not to contaminate this place with silence. Here, people with whom the child speaks unaffectedly like usually caregivers could serve as co-therapists to enhance the likelihood of speaking behavior. In accordance with our assumption, more than half of the parents (56%) described *unknown places* to be significant for their child’s silence. Again, this may demonstrate the strong behavioral inhibition and distress to novelty in children with SM [16]. The fact that the category unknown places was negatively correlated to age while the category places with negative experiences was positively associated to age could indicate a change in important mechanism in the course of development in children with SM. While behavioral inhibition plays a major role in younger children, classical conditioning takes on an increasing role with age and social experience. This result could also comprise important indications for successful intervention for children with SM. It seems to be important to expose young children with a variety of new experiences but at the same time be aware of the child to make positive experience and not to “contaminate” situations with silence. Therefore, gradual exposure in small steps with flanking measures to carefully encourage speaking behavior in children with SM such as defocused communication and child directed interaction [21–24] might be promising treatment approaches. Interestingly, places with a lot of people like *crowds* were mentioned to be an important factor for SM symptomatology by parents with 44%. It has only been described before that SM is linked to places with many people like family gatherings or social events [30]. However, in a study about the comorbidity profile of children with SM compared to children with SAD, it has been found that children with SM show an elevated rate of agoraphobia in adolescence with 27% as compared to 10% in those with SAD [36]. Therefore, agoraphobic tendencies appear to be present in a large proportion of children with SM at all age groups on a subclinical level and some of them may develop into agoraphobia in adolescence. Another aspect of crowds is that they contain publicity without much control over the people that can observe speaking behavior of the child [30]. The aspect of few control over a situation with lots of people may also be reflected in the fact, that none of the patients with SM and comorbid agoraphobia also presented with panic disorder in the study of Gensthaler et al. [16]. Clinically, crowds should be considered as part of exposure therapy. Lastly, a smaller category mentioned by parents were places with *high volume*. Although this characteristic of a place accounted for only 9% compared to all reported categories, it is noticeable that this percentage fits in well with the findings from research on reduced auditory processing in a small subgroup of children with SM [17–20]. Because this category was negatively correlated to age, it might be assumed that developmental delay in auditory processing displays a role in some children with SM characteristics. However, this conclusion is speculative and longitudinal data on the development of children with SM in comparison to typically developing children are needed to draw a concise conclusion on this topic. Furthermore, the naming of this category was associated with the sex of the child and was reported more frequently by parents with boys. So far, there are no data on gender differences in specific SM symptoms available, but developmental delays are more frequently found in boys compared to girls in general, which might also explain for the gender difference here.

In line with past research [31] we had expected *activities with speech demands* and *attention by others* to have an influence on children’s silence. Both categories were mentioned; however, only 19 and 22% of participants, respectively, named these characteristics of an activity as significant for their children’s silence. Past research mainly focused on this kind of situations given the logical link to speech demanding situations and the high comorbidity rate between SM and SAD. This may have led to other important characteristics not being asked in other studies with a closed answering format. The majority of parents (47%) indicated *new activities* to be associated with their child’s silence which again could be linked to an elevated distress to novelty due to high behavioral inhibition [36], and again a negative correlation with age was found for this category. About a fourth of the parents also mentioned *motor activities* (27%) and activities that the child associates with *failure* lead to SM symptomatology. This result fits well with our assumption that challenging activities are associated with the child’s silence and other research that found increased fears of mistakes [20] in children with SM and the influence of stressful activities [30] on their speaking pattern.

Taken together, with the help of a qualitative research design and open-ended questions we were able to confirm findings from past research, and also identified new factors that were previously only indirectly associated with SM. Thus, we were able to replicate already known results and also gain new insights, which we see as a strength of this study. Another strength of the current study is the comparably huge sample size. However, our study also has several limitations to consider. All and above, the anonymous online-based study design comprises some disadvantages. A comprehensive diagnosis with a clinical interview was not possible here, and the inclusion was instead based on a screening questionnaire. We had chosen this approach because it contains a lower threshold for participation and therefore a higher representativeness regarding children with SM symptomatology. Also, the FSSM was proved to be an excellent measure to differentiate almost perfectly between children with SM and children with other disorders as well as typically developing children. However, a comprehensive diagnostic that also includes comorbidities would be desirable and should be the aim of future research. Such an approach would also rule out the possibility that the results were due to comorbidities rather than SM. With our approach, we can also not entirely rule out that other people than parents have filled in the questionnaires. Therefore, an online-based qualitative study is a good starting point to gain some first insights in an understudied phenomenon such as SM and makes it possible to derive hypotheses for further research, but definitely should be completed by laboratory-based hypotheses testing research approaches. Furthermore, girls were overrepresented in our study, and compared to the population, and children with migration background were underrepresented. Therefore, due to disproportionate distribution, some of the correlations between these variables and categories may have failed to meet significance and compared to epidemiological data our sample might not be representative for children and adolescents with SM. It is also possible that the procedure of an online survey specifically targeted parents who have an affinity for the Internet, which may have led to bias. Also, not all parents gave codable answers which might have limited the representativeness as well.

Another criticism is related to the qualitative methodology we used in our study. The process of category building in qualitative content analysis is to some extent influenced by the researchers' subjective views and the categories we defined might not have been perfectly distinct from each other. However, we followed the well-established guidelines and standards for qualitative content analysis to guarantee a maximum of intersubjectivity and trustworthiness for our analysis, which is also indicated by the high inter-rater-reliabilities which are in the range of “almost perfect” according to Cohen.

## Conclusions

To conclude, there are several important aspects of a person, a place, and an activity that should be considered in the individual diagnostics and treatment of children with SM. Children and/or their parents should not only be explored regarding the obvious SM-typical situations such as speech demands and evaluation by others but also with respect to situations that contain other characteristics such as crowds, high volume, or lack of distance. Graduated exposure in small steps may be the treatment of choice to overcome unfamiliar situations and at the same time guarantee experiences of success rather than creating new experiences of perceived failure and contamination with silence. Behavioral treatment approaches that comprise little demands and scaled focus of attention such as defocused communication and child directed interaction could be promising against this background. Future research should also consider subgroups with respect to age and gender, because different aspects of the factors found varied in their relation with these variables.
